# Machine learning for technical skill assessment in surgery: a systematic review

**DOI:** 10.1038/s41746-022-00566-0

**Published:** 2022-03-03

**Authors:** Kyle Lam, Junhong Chen, Zeyu Wang, Fahad M. Iqbal, Ara Darzi, Benny Lo, Sanjay Purkayastha, James M. Kinross

**Affiliations:** Department of Surgery and Cancer, 10th Floor Queen Elizabeth the Queen Mother Building, St Mary’s Hospital, Imperial College, London, W2 1NY UK

**Keywords:** Surgery, Computer science

## Abstract

Accurate and objective performance assessment is essential for both trainees and certified surgeons. However, existing methods can be time consuming, labor intensive, and subject to bias. Machine learning (ML) has the potential to provide rapid, automated, and reproducible feedback without the need for expert reviewers. We aimed to systematically review the literature and determine the ML techniques used for technical surgical skill assessment and identify challenges and barriers in the field. A systematic literature search, in accordance with the PRISMA statement, was performed to identify studies detailing the use of ML for technical skill assessment in surgery. Of the 1896 studies that were retrieved, 66 studies were included. The most common ML methods used were Hidden Markov Models (HMM, 14/66), Support Vector Machines (SVM, 17/66), and Artificial Neural Networks (ANN, 17/66). 40/66 studies used kinematic data, 19/66 used video or image data, and 7/66 used both. Studies assessed the performance of benchtop tasks (48/66), simulator tasks (10/66), and real-life surgery (8/66). Accuracy rates of over 80% were achieved, although tasks and participants varied between studies. Barriers to progress in the field included a focus on basic tasks, lack of standardization between studies, and lack of datasets. ML has the potential to produce accurate and objective surgical skill assessment through the use of methods including HMM, SVM, and ANN. Future ML-based assessment tools should move beyond the assessment of basic tasks and towards real-life surgery and provide interpretable feedback with clinical value for the surgeon.

PROSPERO: CRD42020226071

## Introduction

Accurate and objective performance assessment is a cornerstone of any surgeon’s training. However, despite the wealth of innovation available to the modern-day surgeon, surgeons continue to rely on relatively blunt metrics, such as operative duration, postoperative outcomes, and complication rates in order to track their performance, which fails to truly capture the surgeon’s intraoperative performance. Whilst feedback on intraoperative performance is available from trainers, this tends to be infrequent, unstructured and prone to variation, leaving consistent tracking of performance difficult.

The move to search for more structured and objective methods of assessing intraoperative performance is by no means novel. A wide variety of rating scales (Table 1), such as the Objective Structured Assessment of Technical Skills (OSATS)^1^ are available which allow expert raters to assess surgeons across domains such as flow of operation, tissue handling, or efficiency. These have also been appropriately adapted to specific specialties^2–4^ or to laparoscopic^5^ or robotic platforms^6,7^. Whilst the use of these scales is widespread amongst academic studies, the uptake within clinical practice remains limited. The reasons for this include the need for an expert reviewer, its time consuming and labor-intensive nature and its tendency to rater bias.

**Table 1:** Shared characteristics of Global Rating Scales.

Criteria	OSATS	GOALS	GEARS	R-OSATS	GRITS	ASCRS	M-OSATS	ASSET	BAKSSS	SARMS
Efficiency	X	X	X	X	X	X	X		X	X
Tissue handling	X	X	X	X	X	X	X			X
Instrument handling and knowledge	X			X	X	X	X	X	X	
Flow of operation	X				X	X		X	X	X
Bimanual dexterity		X		X	X			X	X	X
Depth perception		X	X	X	X				X	X
Knowledge of procedure	X				X		X		X	
Autonomy		X	X					X	X	
Use of assistants	X				X		X			

*OSATS* Objective Structured Assessment of Technical Skills^1,54^, *GOALS* Global Assessment Tool for Evaluation of intraoperative Laparoscopic Skills^5^, GEARS Global Evaluative Assessment of Robotic Skills^6^, *R-OSATS* Robotic-Objective Structured Assessment of Technical Skills^7^, *GRITS* Global Rating Index for Technical Skills^55^, *ASCRS* American Society of Colon and Rectal Surgeons Assessment Tool for Performance of Laparoscopic Colectomy^2^, *M-OSATS* Modified Objective Structured Assessment of Technical Skills^56^, *ASSET -* Arthroscopic Surgical Skills Evaluation Tool^3^, *BAKSSS* Basic Arthroscopic Knee Skill Scoring System^4^, *SARMS* Structured Assessment of Robotic Microsurgical Skills^57^.

A potential solution to these issues is the use of ML. ML can be defined as “the scientific discipline that focuses on how computers learn from data”^8^. Once it is trained or designed empirically, it can process the large volume of data available from the modern-day operating room seamlessly and produce rapid, automated, and reproducible feedback without the need for expert reviewers. The ever-increasing availability of computational power has seen ML be applied across numerous disciplines in medicine, with surgery being no exception. ML and artificial intelligence (AI) has been used across diverse applications in surgery ranging from surgical workflow analysis^9^, to autonomous performance of simple tasks^10^, and postoperative mortality risk prediction^11^. This widespread use of ML has led to the development of the field of Surgical Data Science, which aims to improve the quality and value of surgery through data collection, organization, analysis, and modeling^12,13^. Surgical skill assessment is a growing research topic and the last 10 years has seen rapid increase in the use of ML within this field. However, it remains unclear how and to what extent ML can be applied for surgical performance assessment.

Therefore, the aim of this review is to systematically review the literature concerning ML and surgical performance assessment. The aims are primarily to summarize the major ML techniques used to date in surgical skill assessment and to identify the current challenges and barriers in the field; second to understand what the key sources of data used to develop these tools are and the tasks or procedures that have been assessed; and finally, to understand to what extent ML has been successfully employed to assess surgical performance objectively. Through this systematic review, we aim to define future directions and propose new criteria in this emerging field.

## Results

**Fig. 1:**
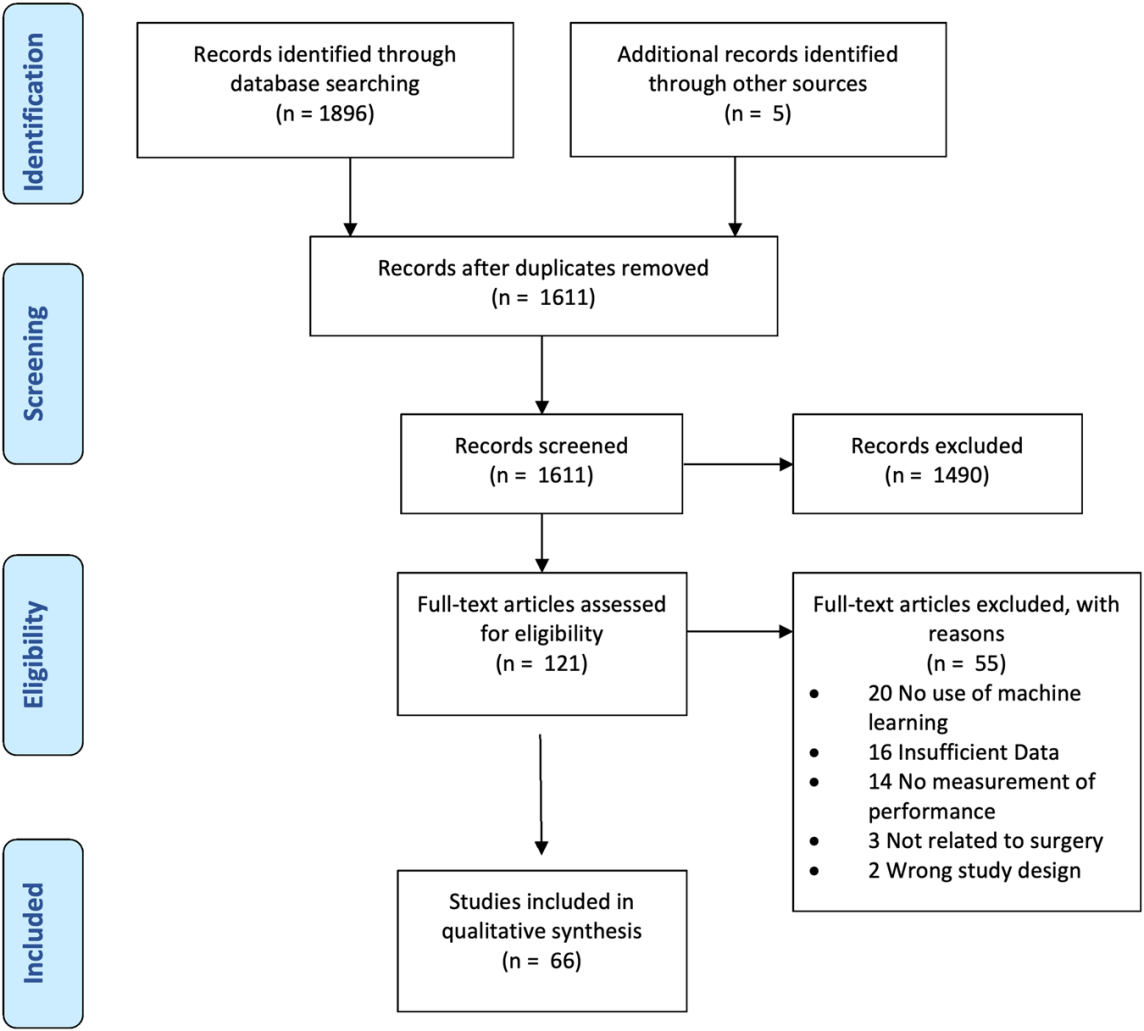
PRISMA flow diagram. Search and study selection process for this review.

**Fig. 2:**
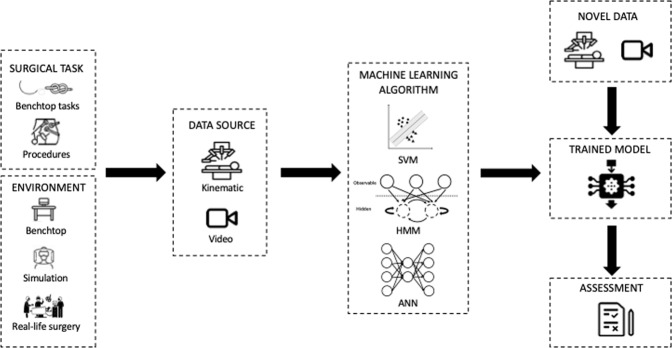
Framework for the technical skill assessment process. Kinematic or video data from differing surgical tasks in a range of environments are recorded and fed to a variety of ML algorithms. The result is the development of a trained model. Novel data can then be fed to these models in order to provide assessment of surgical skill.

The literature search retrieved a total of 1896 studies. A further 5 studies were included through bibliometric cross-referencing. Following title and abstract screening, the full texts of 121 studies were analyzed and 66 studies were found to be eligible for inclusion (Fig. 1). Fig. 2 provides a framework of the technical skill assessment process detailing how novel data can be processed by trained models to provide an assessment of surgical performance. Table 2 provides an overview of all studies included within the review.

**Table 2:** Overview of studies included in the systematic review.

Data	Env	L/R/O	Author	Year	Country	# Trials	# Subjects	Task	Data source	Accuracy
K	B	L	King et al.^58^	2009	UK	7	7	Laparoscopic tissue dissection task	Body sensor network glove	Not specified
K	B	L	Oropesa et al.^59^	2013	Spain, Norway, Netherlands	126	42	Grasp and place (one hand), Coordinated pulling, Grasp and transfer	TrEndo tracking system	71.7–78.2%
K	B	L	Weede et al.^60^	2014	Namibia, Germany	384	96	Knot tying	NDI (EM) aurora tracking system	Not specified
K	B	L	French et al.^61^	2017	US	295	Pool of 98	Peg transfer, Suturing, Circle cutting	EDGE, custom box trainer	2 class: 82.5–87.2%; 3-class: 58.9–65.1%
K	B	L	Dockter et al.^62^	2017	US	91	Pool of 98	Peg transfer, Suturing, Circle cutting	EDGE, custom box trainer	97%
K	B	L	Uemura et al.^63^	2018	Japan	38; 29	28; 29	Suturing	Magnetic tracking sensor on tip of instrument	79%
K	B	L	Oquendo et al.^64^	2018	US, Germany	63	32	Suturing	Ascension trakSTAR 3D EM motion-tracking system	89%
K	B	L	Kowalewski et al.^65^	2019	Germany	99 (knot tying)	28	Suturing, knot tying	Myo armband	MAE OSATS score: 3.7 ± 0.6
K	B	O	Ahmidi et al.^66^	2012	US	378	20	Endoscopic sinus surgery tasks	EM tracker to record endoscope and tool motion. Eye gaze tracker.	88.6–94.6%
K	B	O	Watson^67^	2014	US	48	24	Benchtop Venous anastomosis	Inertial measurement unit	70–83%
K	B	O	Sun et al.^68^	2017	Canada	12	6	Hand tying	Imperial College Surgical Assessment Device	100%
K	B	O	Nguyen et al.^69^	2019	Australia	75, 103	15	Open Suturing, Needle passing, Knot tying	2 wearable inertial processor unit sensors; da Vinci Robot	98.40%
K	B	R	Varadarajan et al.^70^	2009	US	30	8	Suturing, Manipulation, transection, dissection, Needle passing, Knot tying	da Vinci Robot	up to 86%
K	B	R	Reiley et al^71^	2009	US	57	9	Suturing, Manipulation, transection, dissection, Needle passing, Knot tying	da Vinci Robot	95–100%
K	B	R	Tao et al.^72^	2012	US	101	8	Suturing, Manipulation, transection, dissection, Needle passing, Knot tying	da Vinci Robot	LOUO 26.9–59.0% LOSO 94.4–97.4%
K	B	R	Kumar et al.^73^	2012	US	176	12	Suturing, Manipulation, transection, dissection, Needle passing, Knot tying	da Vinci Robot	76.3–83.3%
K	B	R	Ahmidi et al.^74^	2013	US	39; 110	8; 18	Suturing, Manipulation, transection, dissection, Needle passing, Knot tying	da Vinci Robot	91.10%
K	B	R	Forestier et al.^75^	2017	France	103	8	Suturing, Manipulation, transection, dissection, Needle passing, Knot tying	da Vinci Robot	LOSO: SU 93.7%, NP 81.1%, KT 92.5% LOUO: SU 88.3%, NP 75.3%, KT 89.8%
K	B	R	Brown et al.^76^	2017	US	114	38	Suturing, Manipulation, transection, dissection, Needle passing, Knot tying	da Vinci Robot	51.7–75%
K	B	R	Zia et al^77^	2018	US	103	8	Suturing, Manipulation, transection, dissection, Needle passing, Knot tying	da Vinci Robot	Not specified
K	B	R	Wang et al.^78^	2018	US	103	8	Suturing, Manipulation, transection, dissection, Needle passing, Knot tying	da Vinci Robot	LOSO SU 92.5%, NP 95.4%, and KT 91.3%,
K	B	R	Wang et al.^79^	2018	US	103	8	Suturing, Manipulation, transection, dissection, Needle passing, Knot tying	da Vinci Robot	LOSO 96%
K	B	R	Fard et al.^80^	2018	US	75	8	Suturing, Manipulation, transection, dissection, Needle passing, Knot tying	da Vinci Robot	LOSO KT 82.3% SU 89.9% LOUO KT 77.9% SU 79.8%
K	B	R	Ershad et al.^81^	2019	US	84	14	Suturing, Manipulation, transection, dissection, Needle passing, Knot tying	da Vinci Robot	91.05% ± 4.02%
K	B	R	Fawaz et al.^32^	2019	France	103	8	Suturing, Manipulation, transection, dissection, Needle passing, Knot tying	da Vinci Robot	NP 100% SU 100% KT 93.2%
K	B	R	Anh et al.^82^	2020	Australia	103	8	Suturing, Manipulation, transection, dissection, Needle passing, Knot tying	da Vinci Robot	LOSO: 90.17–95.63%
K	B	R	Khalid et al.^83^	2020	US	103	8	Suturing, Manipulation, transection, dissection, Needle passing, Knot tying	da Vinci Robot	RMSE Precision 97%; Recall 98%; RMSE OSATS Precision 77% Recall 78%
K	B	R	Brown et al.^28^	2020	US	740	Not specified	Suturing, Manipulation, transection, dissection, Needle passing, Knot tying	da Vinci Robot	80–98%
K	B	R	Jiang et al.^84^	2017	China	10	10	Peg transfer	Micro Hand S robotic system	Not specified
K	R	R	Hung et al.^18^	2018	US	78	9	Radical Prostatectomy	da Vinci robot	87.20% predicting LOS
K	R	R	Chen et al.^19^	2020	US	68	17	Needle handling/targeting, needle driving, suture cinching	da Vinci robot	77.40%
K	R	O	Ahmidi et al.^23^	2015	US	86	Not specified	Septoplasty	EM sensor on Cottle elevator	91%
K	S	O	Megali et al.^39^	2006	Italy	16	6	Simple surgical tasks	LapSim Basic Skills 1.5 simulator	Not specified
K	S	O	Ahmidi et al.^85^	2012	US	60	20	Endoscopic sinus surgery tasks	Nasal surgery simulator	93%
K	S	O	Poursartip et al.^14^	2017	Canada	26	26	Shoulder arthroscopy	Shoulder arthroscopy simulator	70–95%
K	S	O	Topalli et al.^86^	2019	Turkey	1260	28	Manipulation tasks	Computer-based simulator	86%
K	S	O	Winkler-Schwartz et al.^87^	2019	Canada	250	50	Neurosurgical tumor resection	VR based training platform	90%
K	S	O	Peng et al.^88^	2019	China	420	14	Peg transfer	VR based training platform	96.39%
K	S	O	Siyar et al.^89^	2020	Iran, Canada	115	115	Neurosurgical tumor resection task	VR based training platform	86–90%
K	S	O	Mirchi et al.^16^	2020	Canada	21	21	Anterior Cervical Discectomy	Sim-Ortho simulator	83.30%
V	B	L	Islam et al.^90^	2011	US	35	Aug-19	Peg transfer, knot tying, Suturing, Shape cutting	Endoscopic videos	Not specified
V	B	L	Islam et al.^91^	2013	US, India	52	52	Peg transfer, knot tying, Suturing, Shape cutting	Endoscopic videos	Not specified
V	B	L	Islam et al.^92^	2016	US	156	52	Peg transfer, knot tying, Suturing, Shape cutting	Endoscopic videos	74%
V	B	L	Yamaguchi et al.^27^	2016	Japan	38	38	Peg transfer, knot tying, Suturing, Shape cutting	Endoscopic videos	Not specified
V	B	L	Sgouros et al.^93^	2018	Greece	74	Not specified	Peg transfer, knot tying, Suturing, Shape cutting	Endoscopic videos	96%
V	B	L	Loukas et al.^94^	2020	Greece	64	32	Peg transfer, knot tying, Suturing, Shape cutting	Endoscopic videos	Not specified
V	B	O	Sharma et al.^95^	2014	US, UK	31	16	Suturing, knot tying	Endoscopic videos	93.50%
V	B	O	Zia et al.^96^	2015	US	71	18	Suturing, knot tying	Endoscopic videos	DCT: 85.7–100%; DFT: 91.4–100%
V	B	O	Zia et al^97^	2016	US, UK	71; 33	18; 16	Suturing, knot tying	Endoscopic videos	DCT: 97.4–98.4%; DFT: 95.8–97.7%
V	B	O	Miller et al.^98^	2018	US	70	35	Suturing, knot tying	Endoscopic videos	Not specified
V	B	R	Funke et al.^99^	2019	Germany	103	8	Suturing, Needle passing, Knot tying	Endoscopic videos	LOSO: 95.1%–100.0%.
V	B	R	Gorantla et al.^100^	2019	US	24	12	Urethro-vesical anastomosis	Endoscopic videos	HMM 98.18%, LDA 70%
V	B	R	Gahan et al.^101^	2020	US	23	Not specified	Urethro-vesical anastomosis	Endoscopic videos	65–74%
V	R	L	Jin et al.^21^	2018	US	15	Not specified	Laparoscopic cholecystectomy	Endoscopic videos	Not specified
V	R	R	Baghdadi et al.^20^	2019	US	20	20	Pelvic lymph node dissection from Robot assisted radical cystectomy	Endoscopic videos	83.30%
V	R	R	Lee et al.^24^	2020	South Korea	54	12	Bilateral axillo-breast approach robotic thyroidectomy	Endoscopic videos	83%
V	R	O	Kim et al.^25^	2019	US	99	Not specified	Capsulorhexis	Endoscopic videos	63.4–84.8%
V	R	O	Azari et al.^22^	2019	US	103	9	Hand tie, suturing	Endoscopic videos	Not specified
V	S	O	Zhu et al.^76^	2015	US	23	4	Capsulorhexis	Kitaro simulator	58.3–85.2%
KV	B	L	Rosen et al.^40^	2001	US	8	8	Laparoscopic cholecystectomy, Nissen fundoplication	An instrumented laparoscopic grasper with three-axis force/torque sensor and video	Not specified
KV	B	L	Rosen et al.^38^	2001	US	10	10	Laparoscopic cholecystectomy, Nissen fundoplication	An instrumented laparoscopic grasper with three-axis force/torque sensor and video	87.50%
KV	B	L	Leong et al.^102^	2007	UK	22	11	Point localization	A Polaris infrared tracker on the handle of the laparoscopic instrument and video data	Not specified
KV	B	L	Kelly et al.^103^	2020	US	454	124	Suturing, peg transfer, clipping cutting	Video, kinematic data from EDGE platform	SU 96.9%, PT 87.5%, PC 87.5%, clipping 73.33%
KV	B	O	Zia et al.^104^	2018	United States	74	41	Suturing, Knot tying,	Video and accelerometer data	Video: SU 95.1, KT 92.2 Accelerometer: SU 86.8, KT 78.7%
KV	B	O	Zhang et al.^33^	2020	UK	20–24 per task	8	Positioning task, Path following, Needle insertion	Microsurgical Robot Research Platform	84.7–97.9%
KV	S	O	Bissonnette et al.^17^	2019	Canada	41	41	L3 hemilaminectomy	NeuroVR platform	65.9–97.6%

Data: *K* kinematics, *V* video, *KV* kinematics and video. *Env* environment: *B* benchtop, *S* simulation, *R* real. *L* laparoscopic, *R* robotic, O other. Task: *EM* electromagnetic, *VR* virtual reality. Accuracy: *MAE* mean absolute error, *OSATS* objective structured assessment of technical skill, *LOUO* leave-one-user-out, *LOSO* leave-one-super-trial-out, *SU* suturing, *NP* needle passing, *KT* knot tying, *RMSE* root mean square error, *LOS* length of stay, *DCT* discrete cosine transform, *DFT* discrete fourier transform, *HMM* Hidden Markov Model, *LDA* Linear Discriminant Analysis, *PC* pattern cutting.

### Surgical tasks and environment

48/66 studies assessed the performance of benchtop tasks such as peg transfer, suturing, or knot tying, 10/66 studies used a simulator, and 8/66 studies assessed real-life surgery. Two studies employed the use of animal models in order to conduct procedures such as laparoscopic cholecystectomy. 20/66 studies assessed laparoscopic tasks, 26/66 studies assessed robotic tasks, and the remainder assessed a combination of open tasks such as hand tying or open suturing, or procedures such as arthroscopy^14^ and capsulorhexis^15^. The use of simulators allowed the assessment of more complicated tasks including procedures such as discectomy^16^ or hemilaminectomy^17^. Although studies assessing the performance of real surgery were limited in their number, their proportion has increased since 2018. These studies have investigated procedures across the fields of urology^18–20^, general surgery^21,22^, otolaryngology^23,24^ and ophthalmology^25^. Table 2 details the variety of tasks and environments used in the studies included in this review.

### Data sources

The data sources that form the basis of these ML tools can be divided into kinematic data (40/66) and video or image data (19/66). Seven studies used both kinematic and video data. Kinematic data for the most part was derived from the da Vinci robot (Intuitive USA), but external sensors have been worn by the surgeon or embedded in the instruments to track instrument movement. 10 studies used a simulator. There were few instances of datasets being used on more than one occasion. The most commonly used dataset was the JHU-ISI Gesture and Skill Assessment Working Set (JIGSAWS) dataset^26^ which was used by 10 studies. The size of datasets was small, with 20/66 studies having fewer than 10 participants (Table 2).

### ML methods

**Fig. 3:**
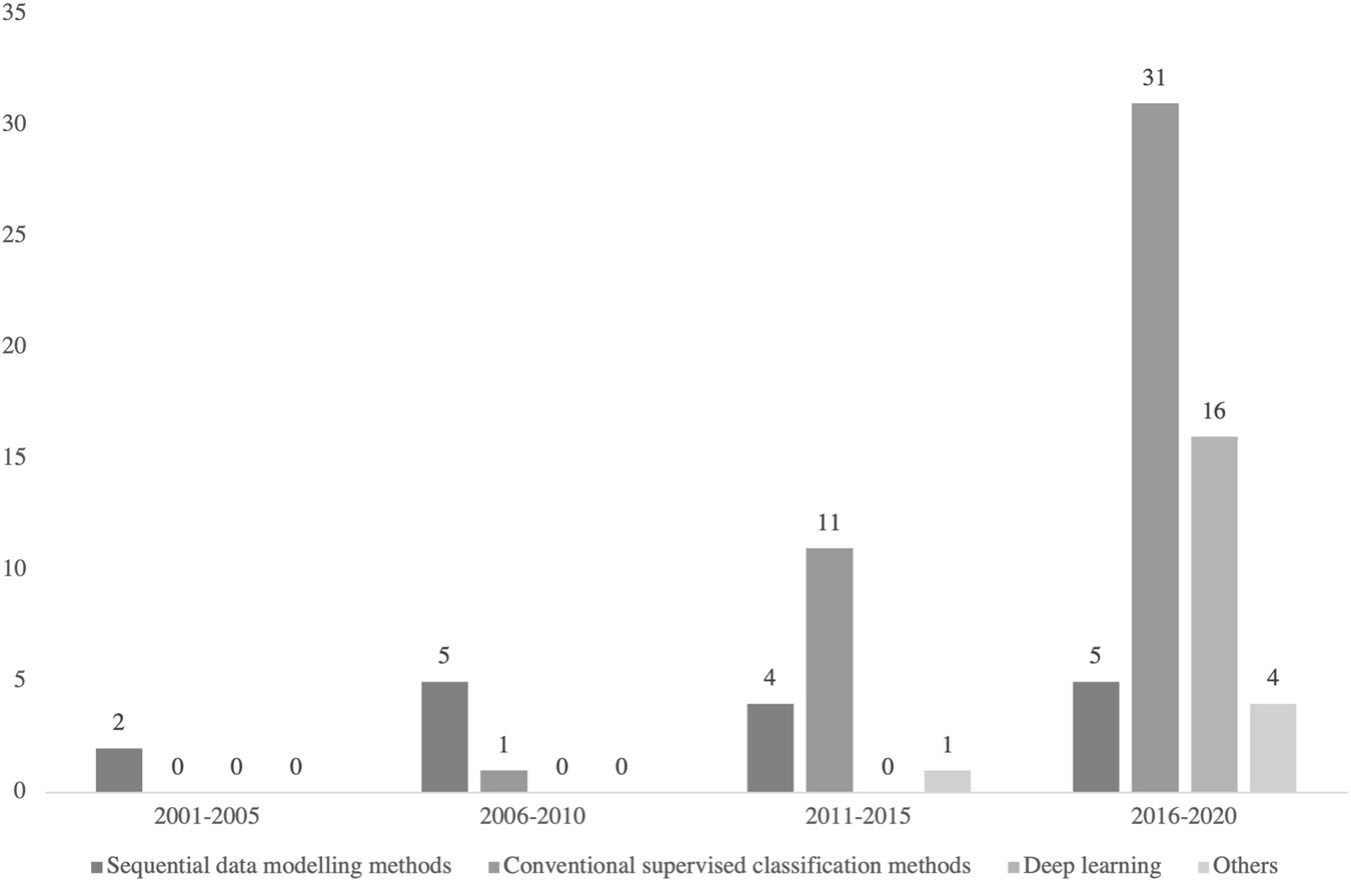
Trends in ML methods used for surgical performance assessment. Graphical depiction of changes in ML methods used for surgical performance assessment between 2001 and 2020.

Whilst a variety of ML methods have been utilized to assess surgical performance, the most common ML methods used were HMM (14/66), SVM (17/66), and ANN (17/66). Incidentally, these three major ML methods coincide with the trends in research within this area; early research focused on the use of HMM before a shift in the field to SVM methods and more recently the use of ANN and deep learning (Fig. 3). Further details of these ML methods and other methods utilized in the studies included in the review are reviewed in Tables 3–7.

**Table 3:** Overview of ML algorithms—sequential data modelling models.

ML Technique	Description	Advantages	Disadvantages	Related Algorithm	References
Hidden Markov Model (HMM)	A probabilistic model which models a series of observable/hidden states and the probability of transition between hidden states. By detecting the transition of the observable states (e.g., bimanual instrument movements), it estimates the most probable sequence of hidden states (e.g., suturing task). The hidden states often represent the surgical manoeuvres, and the metrics can be inferred from the hidden state transitions. Inferred data can then be used to analysis the performance of the surgeon.	1. Low model complexity. 2. Relatively less amount of training data needed. 3. Effective at modeling temporal information.	1. Segmentation of gestures from motion data can be strenuous. 2. Parameter tuning and model development can be time-consuming. 3. Features used in the model are manually defined. 4. Expert knowledge is often required to define the HMM models.	•Maximum Entropy Markov Model. • Markov Random Field. • Conditional Random Fields. • Naïve Bayes	^23,38–40,58,66,68,70–73,88,100,102^
Dynamic Time Warping (DTW)	Algorithm which finds the optimal match between two temporal sequences that vary in time or speed	1. Simple and easy to implement. 2. Highly effective at finding similarities/matches between two sequences.	1. Features need to be manually defined. 2. Can only compare 2 sequences at a time. 3. Long computational time in search for the optimal match.	• Hidden Markov Model	^84,88^

**Table 4:** Overview of ML algorithms: classification methods.

ML Technique	Description	Advantages	Disadvantages	Related Algorithm	References
Support Vector Machine (SVM)	Supervised machine learning method which learns the hyperplane or decision boundary between the classes. The hyperplane is deduced by maximizing the geometric distance between the support vectors of the classes. New data will be projected onto the hyperspace and subsequently classified on the basis of relationship to hyperplane.	1. Can achieve nonlinear classification through kernel. 2. Can be adapted for regression. 3. Easy to understand with low general error. 4. Low inference computational complexity.	1. Difficult to implement for large training data. 2. Difficult in multi-classification problems. 3. Sensitive to missing data, parameters and kernel function selection.	• Support Vector Regression. • Support vector clustering. • Semi supervised SVM	^14,15,17,23,59,61,65,67,71,73,74,80,81,86,87,93,100^
k-nearest neighbors (kNN)	Supervised classification algorithm which groups the points of each class together. During inferencing, the Euclidean distances between the new observed data point and the training data points are calculated. The k-nearest neighbors (i.e., k number of points with the shortest distances to the observed point) are then determined, and the new data point will then be labeled to the class with the highest number of instances in the k-nearest neighbors.	1. No training is required. 2. Low algorithm complexity. 3. Suitable for multi-class problem. 4. Low cost for re-training. 5. Better in processing overlap field of data.	1. Bad performance with high dimensional data. 2. Lazy learning, long inferencing time with large datasets. 3. Sensitive to noise, missing data, and outliers. 4. Requires feature scaling of data. 5. Bad performance when imbalanced sampling datasets.	• k-means clustering	^14,17,77,80,86,87,89,94–96,104^
Naïve Bayes	Supervised classification algorithm based on Bayes Theorem. The simplified from of the Bayes Algorithm—Naïve Bayes is built with the assumption that features are conditionally independent. The class with the highest posterior probability is the outcome of the prediction.	1. Simple logic and robust. 2. Not sensitive to missing data. 3. Performs well when features are close to conditionally independent. 4. Performs well with small datasets.	1. Require conditional independence hypothesis. 2. Tends to not perform as well as more complicated models with larger datasets or correlated features. 3. Require prior probability.	• Bayesian Network. • Maximum a Posteriori. • Maximum likelihood. • Gaussian NB. • Multinomial NB. • Bernoulli NB	^17,86,87^
Decision Tree	Supervised classification algorithm. Data are split repeatedly into subsets and eventually classified at terminal nodes according to logics of nodes along the way.	1. Simple design and interpretable. 2. Suitable for high dimensional data. 3. Low computational power. 4. No domain knowledge or parameter assumptions required. 5. Not sensitive to lost feature. 6. Based on human logics and deterministic.	1. Tends to overfit. 2. Can be unstable as small changes in data can lead to new tree architecture. 3. Calculations can become very complex. 4. Hard to classify temporal sequences. 5. Require preprocessing work. 6. Sequential process and cannot be parallelized.	• Classification and Regression Tree. • Iterative Dichotomiser 3. • C4.5. • Random Forest	^17,64,65^
Random Forest	Supervised classification algorithm. A tree-based algorithm, which combines multiple randomly created decision trees.	1. Reduces overfitting in decision tree and improves accuracy. 2. Flexible to regression problems. 3. Robust to missing data. 4. Fast learning speed.	1. Can require significant computational power. 2. Can be unstable as small changes in data can lead to new tree architecture. 3. Uninterpretable in some feature nodes. 4. High computational cost in inferencing with multiple sequential processes.	• Decision tree	^18,19,62,65,76^
Logistic Regression	Supervised classification algorithm based on the logistic (or sigmoid) function	1. Easy to understand and implement. 2. Fast performance. 3. Good accuracy for simple datasets.	1. Can be easily outperformed by more complex algorithms. 2. Struggles with nonlinear problems. 3. Sensitive to vague features.	• Linear Regression	^1,20,28,61,80^

**Table 5:** Overview of ML algorithms—feature extraction methods.

ML Technique	Description	Advantages	Disadvantages	Related Algorithm	References
Principal Component Analysis (PCA)	Unsupervised linear dimensionality reduction algorithm. It extracts the most significant features with the highest variance in the data.	1. Reduces overfitting. 2. Improves visualization of data. 3. Improves algorithm performance. 4. Removes features which are correlated.	1. Principal components (linear combinations of original features) are abstracted information from data and can be hard to interpret. 2. Sensitive to the scale of features and outliers. 3. Trade-off between information loss and dimension reduction.	• Support Vector Machine. Linear Discriminant Analysis. • Feature selection methods	^27,77,81,82^
Linear Discriminant Analysis (LDA)	Supervised dimensionality reduction and classification algorithm. A statistical method which projects the data onto new axes which maximizes the separability between classes by maximizing the between-class variance and minimizing the within-class variance.	1. Allows for supervised dimensionality reduction with prior knowledge of the classes. 2. Can outperform PCA as dimensionality reduction technique.	1. Not suitable for non-Gaussian samples. 2. Prone to overfitting. 3. The projection space cannot exceed the existing dimensions. 4. Limited by the type of samples.	• Principal Component Analysis	^14,17,59,61,70,87,90,91,100^

**Table 6:** Overview of ML algorithms—clustering methods.

ML Technique	Description	Advantages	Disadvantages	Related Algorithm	References
k-means clustering	Unsupervised iterative clustering algorithm which separates unlabeled data into “k” distinct groupings. Observations sharing similar characteristics are therefore clustered together. New point clustered into one of the K groups based its minimum distance to the center of group. The centers will be recalculated iteratively until convergence. The means of the clusters will then be used to determine the classes of new observed data points.	1. Easy to implement. 2. Low algorithm complexity. 3. Scales to large datasets.	1. Need to assign k, not suitable for some classification requirements. 2. Sensitive to outliers and initial values. 3. Difficult to cluster data of varying sizes. 4. Difficult to implement with high dimensional data. 5. Not suitable for non-convex classification.	• k-nearest neighbors. • Spectral clustering. • Iterative. • Self-organizing maps	^15,60^

**Table 7:** Overview of ML algorithms—deep learning methods.

ML Technique	Description	Advantages	Disadvantages	Related Algorithm	References
Artificial Neural Network (ANN) or Deep Neural Network (DNN)	ANNs are networks of nodes (or neurons) connected to each other to represent data or approximate the functions. DNNs are ANN with many layers (i.e. deep layers). With deep layers and parallel processing of the neurons, ANNs can learn and determine the optimal features from data, and they can be generalized to yield best classification results even with missing data or unseen scenarios.	1. Can achieve high accuracy. 2. Able to model complex and nonlinear problems. 3. Can learn patterns and generalize to handle unseen data. 4. Robust and fault-tolerant to noise.	1. Need large volume of training data. 2. Time-consuming in the training process, and require significant computational power to train complex networks. 3. Difficult to interpret due to its black-box nature. 4. The learning process is stochastic – even training with same data, it may result in different networks.	• Convolutional Neural Networks. • Recurrent Neural Networks	^14,16,21,24,25,32,33,63,65,69,78,79,82,83,99,101,103^
Convolutional Neural Network (CNN)	CNN is an artificial neural network with a “Deep” structure, convolution operation layers and pooling layers. CNN has the ability of representation learning, where it could carry out shift-invariant classification of input information based on its hierarchical structure^105^.	1. Robust. 2. Parallel processing. 3. Learn representative features from data. 4. Can process data with noise and lack of information. 5. Widely used in image classification with high resolution. 6. Pooling can abstract high-level information. 7. Translation invariant (controversial).	1. Time-consuming in the training process, and require significant computational power. 2. Pooling may lose detailed and valued information. 3. Poor performance when input image is of low resolution.	• Multilayer Perceptron. • Recurrent Neural Networks	^21,24,25,32,33,69,78,79,82,99,101^
Recurrent Neural Networks (RNN)	The recurrent neural networks are designed for modeling sequential processes. It takes the current observation together with the output of the network in previous state to generates the output.	1. Parameter sharing mechanism and Turing completeness. 2. Memorizing ability makes it suitable for time-series signal processing involving in semantic analysis, sentiment classification, and language translation.	1. Difficult to train. 2. Imperceptible gradient vanishing problem. 3. Gradient explosion problem, which can be fixed by gradient clipping. 4. Short-term memory issues.	• Long Short-Term Memory. • Gated Recurrent Unit	^33,69,103^

### Assessment and accuracy

52/66 studies reported accuracy rates. The majority of these studies reported accuracy rates of over 80% (Table 2). 31 studies reported accuracy rates of over 90% for at least one task. Accuracy rates for studies assessing the performance of real-life procedures varied between 77.4% and 91.1%. Although accuracy rates reported among these studies were high, these results should be interpreted with caution due to a number of factors.

Firstly, the diverse spectrum of tasks ranging from simple tasks such as peg transfer to complex surgical procedures such as laparoscopic cholecystectomy makes meaningful comparison difficult. Secondly, although all included studies aimed to assess technical surgical performance, the manner in which this was attempted varied between studies. The majority of studies measured surgical performance through the classification of participants into novices or experts. However, other studies aimed to predict scores on global rating scales such as OSATS or GEARS. One study validated the ML-derived assessment metrics against patient outcomes^18^. Moreover, the definitions of novices and experts vary significantly between studies, ranging from the previous number of cases and stage of training to hours of experience. 29/66 studies employed the use of a rating scale such as OSATS in order to determine expertize while 13/66 studies failed to specify how expertize was determined. In addition, definitions of novices varied from medical students with no surgical experience at all to surgeons with less than 5 years of laparoscopic experience^27^.

Finally, cross-validation techniques, a method for assessing the classification ability of the ML model, varied between studies. For example, use of leave-one-user-out (LOUO) validation compared to leave-one-super-trial-out (LOSO) can result in significant differences in accuracy levels. Models validated with the LOUO method tend to achieve lower accuracy scores, when compared with LOSO, as the model is validated on the trials of a surgeon where it has never been trained on. Therefore, the comparison of models with differing cross-validation techniques is problematic. A summary of common cross-validation techniques is presented in Table 8.

**Table 8:** Overview of cross-validation techniques.

Dataset Cross-validation	Description
Hold out	Dataset is randomly split into a training and test set. Can suffer from sampling bias and overfitting to the training set.
k-fold	Data is split into k folds and the data is trained on k-1 folds and tested on the fold that was left out. Process is repeated and the result is averaged. The major advantage is that all observations are used for both training and validation.
Leave-one-user-out (LOUO)	Similar to k-fold validation. In LOUO validation, each surgeon’s trials are used as the test set in turn. Repeated until each surgeon’s trials are used for testing.
Leave-one-super-trial-out (LOSO)	Also a variation on k-fold validation. In LOSO validation, a trial from each surgeon’s set of trials is used as the test set. This process is repeated and the result is averaged. This tends to achieve better results compared to LOUO as the algorithm can learn on all surgeons’ trials.
Bootstrapping	Bootstrapping is similar to k-fold validation but resamples with replacement such that the new training datasets will always have the same number of observations as the original dataset. Due to replacement, bootstrapped datasets may have multiple instances or completely omit the original cases.

### Quality Assessment

The mean MERSQI score was 11.6. Scores ranged from 10.5 to 14.5. The majority of studies were designed as single group studies without randomization, single center in nature and had outcomes of skills and behaviors limiting their maximum possible score. The full table of results can be found in the Supplementary Data 2.

## Discussion

This systematic review demonstrates the variety of ML techniques used in the assessment of technical skill in surgery. A total of 66 studies employed the use of ML in order to perform technical skill assessment in surgery. The most commonly used ML models were HMM, SVM, and ANN. However, of the studies included in this systematic review which took place in 2019 or later, half involved the use of neural networks, which reflects its increase in popularity.

31 studies reported accuracy rates of over 90% on determining performance on at least one task, highlighting the promise ML-based surgical performance assessment has to offer. This review demonstrates that ML-based surgical performance assessment has the potential to be incorporated into surgical training in order to deliver accurate performance assessment which is objective, reproducible and not resource intensive. This technology could allow surgical trainees to gain access to regular and consistent feedback, allowing them to track and progress up their learning curves more rapidly. Moreover, the benefits of ML-based surgical assessment tools could extend beyond surgical trainees; for example, allowing certifying bodies to deem surgical competence or assessing how surgeons perform with novel technologies or techniques in the operating room.

Despite the significant promise that this field offers, this review highlights that ML-based surgical assessment tools are still within their relative infancy and that a tool, which can be delivered into clinical practice appears distant. We highlight three significant barriers to progress and suggest key future research goals.

### Focus on basic tasks

The majority of studies included in our systematic review focused on the assessment of performance in basic benchtop tasks such as suturing, peg transfer, and knot tying. Whilst the reported accuracy of determining novices and experts at these tasks were high, the translation of these techniques into life surgery is called into question. Real-life surgery has significant challenges to overcome when compared to an artificial benchtop environment. Algorithms have to contend with less predictable kinematic data as well as video which can be contaminated with blood and surgical smoke. Therefore, the applicability of techniques used in these environments may have limited value when employed in life surgery.

Moreover, the value of determining novices and experts from these relatively trivial tasks may be limited beyond those initially learnt on laparoscopic or robotic platforms. Classification of surgeons into novices and experts may be purely a surrogate of familiarity with the platform rather than of actual surgical skill. In addition, it is questionable whether the measurement of performance on these tasks truly determines technical surgical skill rather than simply the dexterity of the participant. In one study, there were no statistically different objective performance indicators between robotic experts and training specialists, defined as non-surgeons with significant experience in benchtop robotic tasks^28^. It must be noted that multiple studies attempt to classify participants into novices, intermediates and experts. Efforts to differentiate between those with moderate levels of experience to experts will likely have more clinical transferability compared to studies, which aim to classify participants with significant disparities in ability, such as medical students against expert surgeons. Therefore, whilst the use of basic tasks is an obvious first step for those aiming to develop these ML tools due to the relative ease and speed of data collection, it must be recognized that the clinical value of such studies may be limited.

### Lack of standardization of methods

Across the 66 studies reviewed in our systematic review, there is significant variation amongst the studies carried out. Whilst the majority of studies compared novices to experts, definitions of novices and experts varied significantly. Novices varied from medical students with no surgical experience to residents on a defined surgical training programme whilst the definition of expert ranged from 50 cases to 1000 cases. While some studies classified participants against a ground truth of an expert-rated scale such as OSATS or GEARS, the majority of studies based expertize on hours of training or the number of cases performed. Some studies based expertize level on the stage of training which may not be an accurate representation of expertize level (for example, due to varying levels of exposure to robotic platforms), while other studies entirely failed to state how expertize was determined.

In combination with the diverse range of tasks and different cross-validation techniques employed in these studies, the comparison of methods used to assess performance is challenging. Some success has been achieved with the JIGSAWS dataset^26^, an open-source annotated dataset of eight surgeons across three expertize levels performing a total of 103 basic robotic benchtop trials. The use of this dataset by multiple research groups has allowed the comparison of assessment techniques on a benchmark dataset. However, beyond the JIGSAWS dataset, we have found few studies have compared results across the same datasets. The majority of studies within our review present methods based on original data with varying methodology rendering comparison difficult.

### Lack of data

The datasets within this systematic review were small in nature with 20/66 studies comprising of fewer than 10 participants. In addition, the majority of data obtained from these studies were not open-source and therefore datasets were not reused across different groups. There is, however, increasing momentum for the sharing of datasets such as *m2cai2016-tool*^29^ released for the tool presence detection challenge at M2CAI 2016 and datasets used in the EndoVis challenges^30^. The increasing availability of open-source datasets will allow not only the benchmarking of results but also improved training and performance of models, as well as encouraging a global effort towards publishing more datasets.

Whilst inadequate amount of data is a common problem amongst ML communities, acquisition of real-life surgical data poses its unique set of challenges. There is a lack of digitization and infrastructure across operating rooms meaning that those collecting data such as operative video are, for the most part, in the minority. Ultimately, for ML applications in surgery to flourish, a paradigm shift in the operating room towards large-scale collection of surgical data is needed in order to facilitate these applications. However, implementing these systems are not without issue and the surgical data science community continues to grapple with both the technical and ethical hurdles to its adoption^13,31^.

### Moving forward

Studies investigating performance assessment in surgery must move away from basic benchtop tasks and towards assessment of real-life surgery. However, the increasingly popular use of deep learning architectures requires large volume of intraoperative data. The priorities must be to ensure operating rooms are appropriately digitized and have the infrastructure to both collect and share intraoperative data. Not only will the sharing of these datasets improve the development of ML models and allow comparison of techniques but it will also encourage collaboration between groups to further research in this area. This will solve not only issues associated with the use of ML in surgical performance assessment but also issues across the whole field of surgical data science and the wider application of ML to surgery. Encouragingly, efforts have been made by the surgical data science community in order to identify the challenges and research targets associated with widespread data acquisition in the operating room and data sharing^13,31^. It is only through this that datasets can be acquired and utilized at scale.

Future studies should aim to standardize methodology such that meaningful comparison can be made. Individual studies with varying skill levels of participants performing a wide variety of tasks are unlikely to be impactful when compared to studies with standardized methodology ideally on shared open-source datasets. Furthermore, skill assessment in surgery must move beyond a simplistic binary classification. The clinical applicability of being classified as a novice as opposed to an expert is limited; it is more important for trainees to understand why they have been classed as a novice than just to know that they have been classed as such. The focus within this field must move towards explainable techniques. Class activation maps are able to inform the surgeon which aspect of the task has weighted their classification towards a novice or expert, allowing the trainee to understand which part of the task they should look to improve upon in the future^32,33^. Not only must future performance assessment tools be accurate, but they must identify targets of improvement which are interpretable to the surgeon. The future performance assessment tool must move beyond a novice vs expert classifier and towards a clinically applicable tool, which can continuously assess surgeon performance and therefore advance surgeons up their learning curves more rapidly and maintain their performance.

The significant promise lies in the emergence of novel frameworks within the ML community which may be able to counter the problems faced by neural networks, such as the large volume of training data required (Table 7). Generative adversarial networks (GAN), through the use of two competing neural networks, are able to generate novel data with the same features as the training data^34^. Its application has seen huge popularity in the fields of AI art and the creation of new photographs which appear superficially authentic to human observers. The application of GAN to ML-based surgical assessment could address issues with insufficient training data, which is often a limiting step within the development of these tools. Transformer networks^35^, an encoder-decoder architecture based on attention layers, have rapidly gained popularity within the field of Natural Language Processing due to its power for sequential modeling. ML-based surgical assessment tools could apply transformers and their capability to model temporal relationships to model surgical phase transitions. Clinicians must work in conjunction with ML scientists so that advances within ML development can be capitalized upon and applied within the field of ML-based surgical assessment. Furthermore, ML scientists must have an understanding of the surgical challenges and needs that they are trying to solve. It is only through a mutual awareness of each others’ fields that ML-based surgical assessment can advance.

Finally, the development of ML-based surgical assessment tools is not limited to the technical challenges alone. The future use of ML for the purposes of surgical technical skill assessment may bring wider challenges. ML-based assessment of future surgical teams may challenge the rights of privacy for the surgeon and their team. Not only are there fears from surgeons that they will be constantly watched, but there are also concerns that such systems may influence surgeon’s behaviors. In addition, it is unclear what the rights of the future surgeon to opt-out are as well as the implications of doing so. Finally, it is unclear what the role of such systems may play in the role of determining surgical error. Whilst ML-based performance assessment tools may allow rapid, reproducible, and automated performance assessment and in doing so accelerate surgical education, we must also pre-empt the potential wider challenges of implementing such tools into clinical practice. We must look, not only at the development of these performance assessment tools, but also the challenges associated with their deployment. Ultimately, for research into ML-based performance assessment tools to be worthwhile they must be leveraged such that they can make the transition from benchtop to bedside.

## Conclusions

Despite research spanning 20 years, there is still significant progress to be made in the use of ML for technical skill assessment. The use of ML has the opportunity to allow surgeons to track their performance accurately, objectively, and reliably. Numerous ML methods have been utilized to assess surgical skill; however, the comparison of such techniques is difficult due to the wide variety of datasets, tasks, and study participants. We identify three key barriers to progress in the field: (1) a focus on basic benchtop tasks; (2) the lack of standardization between studies; (3) the lack of available datasets for the purpose of surgical assessment. Future efforts in the field must focus on moving beyond basic benchtop tasks and towards the assessment of real-life surgery which is interpretable and of clinical value for the surgeon. For this to be successful, operating rooms must adapt to allow intraoperative data to be acquired at scale and subsequently shared.

## Methods

This systematic review was conducted according to the Preferred Reporting Items for Systematic Reviews and Meta-Analyses statement (PRISMA)^36^. The systematic review was also registered on the International Prospective Register of Systematic Reviews (PROSPERO ID: CRD42020226071).

### Search Strategy and Databases

A comprehensive literature search was conducted using Medline (via Ovid), Embase, Web of Science, and the IEEEXplore database to account for technical papers. Example search terms included ‘machine learning’ and ‘artificial intelligence’ in addition to ‘surgical skill’, ‘surgical performance’, and ‘surgical assessment’. The full Medline, Embase, Web of Science, and IEEEXplore search strategies can be found in Supplementary Data 1. Free-text words were combined using Boolean operators, in addition to medical subject headings terms (MeSH). The search was performed in consultation with a professional librarian at Imperial College London in December 2020.

All identified studies were uploaded to Covidence, a Cochrane-supported systematic review package tool. Initial screening was independently conducted by two investigators (KL and FMI) to determine if the eligibility criteria were met. Discrepancies were discussed and resolved either by consensus or by a third reviewer. Studies that met the inclusion criteria underwent full-text screening. In addition, supplemental references were examined for additional relevant articles.

### Study selection criteria and outcome measures

Studies published including the primary and secondary outcomes as detailed below were included. No language restrictions were applied. Inclusion criteria included any study that used ML to examine performance assessment of either a real-life operative procedure or a surgical benchtop task. Exclusion criteria included any study that did not assess performance or did not use a ML technique. The last search was conducted in December 2020. Studies with inadequately published data with regards to the primary and secondary outcome measures were also excluded.

### Data extraction

The primary outcome of this systematic review was to detail the ML techniques used in technical skill assessment in surgery and identify the current challenges and barriers in the field. Secondary objectives were to understand the types of data employed by these ML techniques, determine the procedures and tasks which have been investigated in these studies and determine the current accuracy of existing ML models used for surgical skill assessment. We determined real-life studies as studies that utilized data taken from real-life surgery, simulator studies as studies, which recorded data without the need for external sensors (able to automatically generate kinematics or metrics without noise and the need for preprocessing), and benchtop studies as any study that did not satisfy the previous two criteria.

All study characteristics and outcome measures were independently extracted by two investigators (KL and FMI). Discrepancies were discussed and resolved either by consensus or by a third reviewer.

### Quality Assessment (Risk of Bias)

Quality assessment was conducted through the use of the Medical Education Research Study Quality Instrument (MERSQI)^37^. The 10-item tool assesses 6 domains, each with a maximum score of 3, (1) study design, (2) sampling, (3) type of data, (4) validity of evaluation instrument, (5) data analysis, (6) outcomes. Scores range from 0–18. Quality assessment was assessed by one reviewer and validated by a second.

### Overview of ML methods

HMM can be seen as a probabilistic method to predict the unobservable sequence (usually the underlying tasks, the movement orders of instruments, etc.) based on the probability of the sequence of occurrence of observable information (such as kinematic data of the surgical instruments, visual features, force exerted). In surgical skill assessment, HMM will enable the researcher to infer the underlying sequences of surgical tasks, instrument motion trajectories, etc., from the observable information captured during the operations and which can be used to distinguish and quantify the surgical dexterities of surgeons. For example, for the same surgical task, such as suturing, a novice may take more steps and time (i.e. a longer sequence of instrument movements) compared to an expert surgeon. A classic example can be found in Rosen et al.^38^.

In early articles, HMM is widely used as the training method to assess surgical skill. HMM were applied to estimate the underlying surgical maneuvers from the observable kinematic/video data from the system when the surgeon participant performed surgical training tasks, and the participant’s training skill level was then deduced from the estimated data^38–40^. Although accuracy within this period achieved over 80%, the use of HMM failed to demonstrate sufficient benefit for it to be employed on a wider scale. However, the early use of HMM had led to the growing interests in the use of ML for the purpose of surgical skill assessment. The use of HMM declined at the start of the 2010s with the rise in popularity of ML methods such as SVM.

SVM^41,42^ is a supervised ML method based on the Vapnik-Chervonenkis Dimension theory and structural risk minimization principle^43^ to address linear and nonlinear classification problems, which denote the distribution of the input dataset. Generally, the use of SVM classifiers consist of the training stage, validation stage, and test/prediction stage. The SVM classifier relies on the multi-dimensional handcrafted features and metrics relevant to the tasks of interest derived from original signals, such as bio-signal^44^, video^45^, kinematic data^46^. Such features include energy-based metrics^14^ (which include total work, the sum of the changes in potential energy, and the sum of the changes in kinetic energy when performing a specific task), computer vision-based features^15^ (such as duration, size, centrality, circularity, and motion stability), and other measurable indexes (such as the position, angle, and force application of instruments and volume of simulated tissue removed^17^. These features vectors or matrix are often linearly inseparable. Hence, conventional linear classifiers, such as Linear Discriminant Analysis, are not able to classify the tasks based on these feature vectors. However, the SVM classifier maps the original features from a low dimensional space to a higher dimensional space nonlinearly and transform the nonlinear problem into a linear separable one, so that the classification boundary or the ‘hyperplane’ (in the higher dimensional space) of the original features matrix can be determined by maximizing the margin between the key feature points (i.e. the support vectors).

In essence, it avoids the traditional process from induction to deduction, realizes the efficient “transductive reasoning” from training samples to prediction samples (hence, maximizing the margin between the support vectors), and greatly simplifies common classification and regression problems. Therefore, it can yield high classification accuracy even with relatively small training data samples. However, since SVM calculates support vectors by quadratic programming, which involves the calculation of an m-order matrix, the storage and calculation of the matrix requires significant computational power and machine memory. In addition, computing resource will increase with the number of samples and therefore SVM can be difficult to train with large-scale training samples. SVM can be sensitive to missing data, parameters, and kernel function selection which has limited its widespread applications in big data analytics.

ANN are inspired by the biological information processing mechanism of the human neural system. An ANN consists of a network of interconnected nodes (or neurons) to simulate the functions of the soma, dendrite, and axon of the neurons and the synaptic connections between the neurons to realize strategy representation or function approximation. ANN can learn and deduce the optimal approximation of highly complex nonlinear functions, given its ability to learn from the data. Common topological structures include multi-layered feed forward network, feedback network, recurrent neural network and competitive neural network^47^.

The concept of ANN is to imitate the human’s cognitive abilities. Like the biological neurons in the human brain, neurons in ANN can gather information from multi-inputs (i.e. from their connected neurons or stimuli), process the information and output signals to its connected neurons (or the classification results). Both biological neural networks (BNN) and ANN can receive signals (electro-chemical signals in BNN, data signals in ANN), and release the processed signals to the connected neurons. Unlike BNN, ANN are designed with layered structures, where signals can be gathered and passed between layers but not across layers. Signals which are passed between neurons will be amplified or attenuated with the synaptic weights, and each neuron will activate or deactivate based on the weighted synaptic signals it receives. In other words, ANN learns and memorizes information through adjusting the synaptic weights between neurons. Deep learning or deep neural network (DNN) refers to ANN with many layers of neurons, and increasing the number of layers and neurons will increase the inferencing ability of the ANN, especially in highly complex nonlinear problems.

The last few years have seen increasing numbers of applications of ANN in the field of surgical skill assessment, which can be categorized into conventional ANN (used mostly in earlier research), and DNN (used in recent research). The conventional sequential modeling-based ML methods, such as SVM, require the design of optimized data preprocessing functions, feature symbolization or quantification and feature selection processes which are a very complex process and require expert knowledge. In contrast, the new end-to-end^48,49^ method framework, (i.e. the DNN method), can learn the optimal features directly from the data and extract high-level abstract information, which will lead to high classification accuracy. This framework is gradually becoming the standard approach in ML. The emergence of different deep network topologies, such as Generative adversarial network (GAN)^50^ (which is designed for addressing insufficient available data sources for training the neural network), Convolutional neural network (CNN)^51^ (which is designed for learning the optimal features from data, especially for vision-based applications), Recurrent Neural Network (RNN)^52^ and Long-Short-Term Memory (LSTM)^53^ (which are designed for time series classification tasks), coupled with ever-increasing computational power due to the advances in the semiconductor industry, offer great potential in the development of objective surgical skill assessment tools.

### Reporting Summary

Further information on research design is available in the Nature Research Reporting Summary linked to this article.

## Supplementary information

Supplemental material

Nature Reporting summary

## Data Availability

The datasets generated during and/or analyzed during the current study are available from the corresponding author on reasonable request.
