# Supplementary material for: Machine learning for technical skill assessment in surgery: a systematic review
Source: NPJ Digit Med. 2022 Mar 3;5:24. doi: 10.1038/s41746-022-00566-0 (PMC8894462; doi:10.1038/s41746-022-00566-0)
Supplement: Supplementary file 1 — Supplemental material [file 41746_2022_566_MOESM1_ESM.pdf]

SUPPLEMENTARY DATA 1: SEARCH STRATEGY

Databases: MEDLINE (via Ovid), EMBASE, Web of Science and IEEEXplore

<u>MEDLINE = 429</u>

“Deep learning” [MESH] or "Machine Learning"[Mesh] OR "Artificial Intelligence"[Mesh] OR "Neural Networks (Computer)"[Mesh] OR "Support Vector Machine"[Mesh] OR “Algorithms” [MESH] OR “Bayes theorem” [MESH] OR Markov Chains[MESH] OR Machine learning OR Artificial Intelligence OR Naive Bayes OR Neural network* OR support vector machine OR SVM OR random forest* OR boosting OR adaboost OR deep learning OR (deep or convolutional or bayesian) network* OR bayes classifier OR machine intelligence OR computational intelligence OR or (nearest adj1 (neighbor or neighbour)) or “multilayer perceptron” OR computer vision OR hidden markov mode* OR hmm

AND

Surgical performance OR surgical skill OR surgical assessment OR technical skill OR technical performance OR technical assessment OR surgeon* adj3 performance OR surgeon* adj3 assess* OR surgeon* adj3 skill

<u>Embase = 388</u>

(“Deep learning” [mesh]" OR "Machine Learning"[mesh]" OR "Artificial Intelligence" [mesh]" OR "artificial Neural Network[mesh]" OR convolutional neural network [mesh]"

"Support Vector [mesh]" " OR Algorithm [mesh] OR " Bayes theorem" [mesh] OR markov chain[mesh] or OR Machine learning OR Artificial Intelligence OR Naive Bayes OR Neural network* OR support vector machine OR SVM OR random forest* OR boosting OR adaboost OR deep learning OR (deep or convolutional or neural or bayesian) network* OR bayes classifier OR machine intelligence OR computational intelligence OR or (nearest adj1 (neighbor or neighbour)) or "multilayer perceptron" OR computer vision OR hidden markov mode* OR hmm

AND

Surgical performance OR surgical skill OR surgical assessment OR technical skill OR technical performance OR technical assessment OR surgeon* NEAR/3 performance OR surgeon* NEAR/3 assess* OR surgeon* NEAR/3 skill

Web of Science = 595

TS = (Skill Assess* OR Performance assess* OR technical assess*) 445,970

AND

TS = (surgeon OR surgery OR surgical) 1,568,312

AND

TS = (Deep Learning OR Machine learning Or Artificial intelligence OR neural network* OR computer vision OR support vector machine OR Bayesian OR markov chain* OR hidden markov model* OR adaboost) 544,866

1,2,3 = 595

IEEEExplore = 484

(("Abstract": performance OR "Abstract": skill OR "Abstract": assess) AND ("Abstract": surgeon OR "Abstract": surgery OR "Abstract": surgical) AND (machine learning OR automat OR artificial intelligence OR computer vision))

SUPPLEMENTARY DATA 2: MERSQI QUALITY ASSESSMENT

Author	Year	Study Design /3	Sampling /3	Type of Data /3	Validity of evaluation instrument /3	Data analysis /3	Outcomes /3	Total/ 18
**BENCHTOP**								
**Rosen et al[76]**	2001	1	2	3	0	3	1.5	**10.5**
**Rosen et al[77]**	2001	1	2	3	0	3	1.5	**10.5**
**Leong et al[78]**	2007	1	2	3	2	3	1.5	**12.5**
**Varadarajan et al[30]**	2009	1	2	3	0	3	1.5	**10.5**
**Reiley et al[31]**	2009	1	2	3	0	3	1.5	**10.5**
**King et al[18]**	2009	1	2	3	0	3	1.5	**10.5**
**Islam et al[58]**	2011	1	2	3	0	3	1.5	**10.5**
**Tao et al[32]**	2012	1	2	3	0	3	1.5	**10.5**
**Kumar et al[33]**	2012	1	3	3	3	3	1.5	**14.5**
**Ahmidi et al[26]**	2012	1	2	3	0	3	1.5	**10.5**
**Islam et al[59]**	2013	1	2	3	0	3	1.5	**10.5**
**Ahmidi et al[34]**	2013	1	2	3	0	3	1.5	**10.5**
**Oropesa et al[19]**	2013	1	2	3	0	3	1.5	**10.5**
**Sharma et al[64]**	2014	1	2	3	2	3	1.5	**12.5**
**Weede et al[20]**	2014	1	2	3	1	3	1.5	**11.5**
**Watson[27]**	2014	1	2	3	1	3	1.5	**11.5**
**Zia et al[65]**	2015	1	2	3	3	3	1.5	**13.5**
**Islam et al[60]**	2016	1	2	3	3	3	1.5	**13.5**
**Yamaguchi et al[61]**	2016	1	2	3	0	3	1.5	**10.5**
**Zia et al[66]**	2016	1	2	3	2	3	1.5	**12.5**
**Dockter et al[22]**	2017	1	2	3	0	3	1.5	**10.5**
**Forestier et al[35]**	2017	1	2	3	2	3	1.5	**12.5**
**Jiang et al[46]**	2017	1	2	3	0	3	1.5	**10.5**
**French et al[21]**	2017	1	2	3	2	3	1.5	**12.5**
**Brown et al[36]**	2017	1	2	3	3	3	1.5	**13.5**
**Sun et al[28]**	2017	1	2	3	0	3	1.5	**10.5**
**Zia et al[80]**	2018	1	2	3	3	3	1.5	**13.5**

**Zia et al[37]**	2018	1	2	3	2	3	1.5	**12.5**
**Miller et al[67]**	2018	1	2	3	0	3	1.5	**10.5**
**Uemura et al[23]**	2018	1	2	3	0	3	1.5	**10.5**
**Sgouros et al[62]**	2018	1	2	3	0	3	1.5	**10.5**
**Oquendo et al[24]**	2018	1	2	3	3	3	1.5	**13.5**
**Wang et al[38]**	2018	1	2	3	2	3	1.5	**12.5**
**Wang et al[39]**	2018	1	2	3	1	3	1.5	**11.5**
**Fard et al[40]**	2018	1	2	3	1	3	1.5	**11.5**
**Nguyen et al[29]**	2019	1	2	3	0	3	1.5	**10.5**
**Gorantla et al[69]**	2019	1	2	3	0	3	1.5	**10.5**
**Ershad et al[41]**	2019	1	2	3	0	3	1.5	**10.5**
**Kowalewski et al[25]**	2019	1	2	3	2	3	1.5	**12.5**
**Funke et al[68]**	2019	1	2	3	0	3	1.5	**10.5**
**Fawaz et al[42]**	2019	1	2	3	0	3	2	**11**
**Anh et al[43]**	2020	1	2	3	0	3	1.5	**10.5**
**Khalid et al[44]**	2020	1	2	3	0	3	1.5	**10.5**
**Loukas et al[63]**	2020	1	2	3	0	3	1.5	**10.5**
**Zhang et al[81]**	2020	1	2	3	0	3	1.5	**10.5**
**Kelly et al[79]**	2020	1	3	3	2	3	1.5	**13.5**
**Gahan et al[70]**	2020	1	2	3	2	3	1.5	**12.5**
**Brown et al[45]**	2020	1	2	3	1	2	1.5	**10.5**
**SIMULATION**								
**Megali et al[50]**	2006	1	2	3	0	3	1.5	**10.5**
**Ahmidi et al[51]**	2012	1	2	3	2	3	1.5	**12.5**
**Zhu et al[83]**	2015	1	2	3	3	3	1.5	**13.5**
**Poursartip et al[52]**	2017	1	2	3	2	3	1.5	**12.5**
**Topalli et al[53]**	2019	1	2	3	0	3	1.5	**10.5**
**Winkler-Schwartz et al[54]**	2019	1	2	3	0	3	1.5	**10.5**
**Peng et al[55]**	2019	1	2	3	1	3	1.5	**11.5**
**Bissonnette et al[82]**	2019	1	3	3	1	3	1.5	**12.5**
**Siyar et al[56]**	2020	1	2	3	0	3	1.5	**10.5**

**Mirchi et al[57]**	2020	1	2	3	0	3	1.5	**10.5**
**REAL**								
**Ahmidi et al[49]**	2015	1	2	3	0	3	1.5	**10.5**
**Jin et al[71]**	2018	1	2	3	2	3	1.5	**12.5**
**Hung et al[47]**	2018	1	2	3	2	3	3	**14**
**Baghdadi et al[72]**	2019	1	2	3	2	3	1.5	**12.5**
**Kim et al[74]**	2019	1	2	3	3	3	1.5	**13.5**
**Azari et al[75]**	2019	1	2	3	3	3	1.5	**13.5**
**Lee et al[73]**	2020	1	2	3	2	3	1.5	**12.5**
**Chen et al[48]**	2020	1	2	3	0	3	1.5	**10.5**
